# Axonal Membranes and Their Domains: Assembly and Function of the Axon Initial Segment and Node of Ranvier

**DOI:** 10.3389/fncel.2017.00136

**Published:** 2017-05-09

**Authors:** Andrew D. Nelson, Paul M. Jenkins

**Affiliations:** ^1^Department of Pharmacology, University of Michigan Medical SchoolAnn Arbor, MI, USA; ^2^Department of Psychiatry, University of Michigan Medical SchoolAnn Arbor, MI, USA

**Keywords:** axon initial segment, nodes of Ranvier, ankyrin-G, spectrin, cytoskeleton, excitable membrane domains, sodium channels

## Abstract

Neurons are highly specialized cells of the nervous system that receive, process and transmit electrical signals critical for normal brain function. Here, we review the intricate organization of axonal membrane domains that facilitate rapid action potential conduction underlying communication between complex neuronal circuits. Two critical excitable domains of vertebrate axons are the axon initial segment (AIS) and the nodes of Ranvier, which are characterized by the high concentrations of voltage-gated ion channels, cell adhesion molecules and specialized cytoskeletal networks. The AIS is located at the proximal region of the axon and serves as the site of action potential initiation, while nodes of Ranvier, gaps between adjacent myelin sheaths, allow rapid propagation of the action potential through saltatory conduction. The AIS and nodes of Ranvier are assembled by ankyrins, spectrins and their associated binding partners through the clustering of membrane proteins and connection to the underlying cytoskeleton network. Although the AIS and nodes of Ranvier share similar protein composition, their mechanisms of assembly are strikingly different. Here we will cover the mechanisms of formation and maintenance of these axonal excitable membrane domains, specifically highlighting the similarities and differences between them. We will also discuss recent advances in super resolution fluorescence imaging which have elucidated the arrangement of the submembranous axonal cytoskeleton revealing a surprising structural organization necessary to maintain axonal organization and function. Finally, human mutations in axonal domain components have been associated with a growing number of neurological disorders including severe cognitive dysfunction, epilepsy, autism, neurodegenerative diseases and psychiatric disorders. Overall, this review highlights the assembly, maintenance and function of axonal excitable domains, particularly the AIS and nodes of Ranvier, and how abnormalities in these processes may contribute to disease.

## Introduction

**Figure 1:**
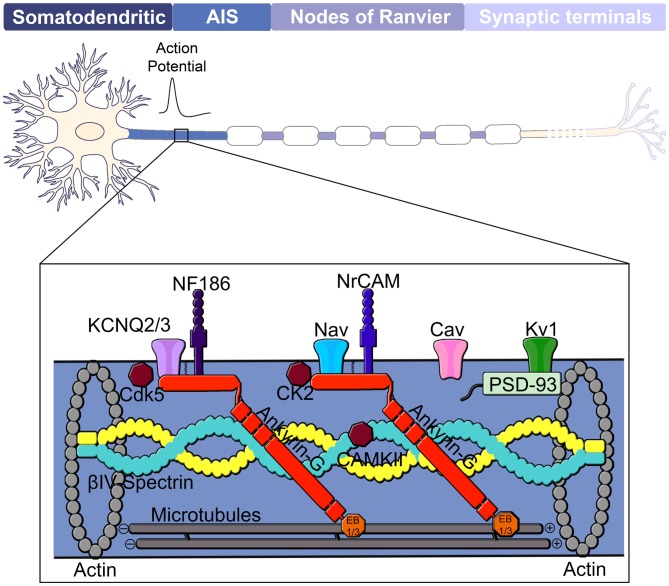
**Domain organization of a neuron.** The neuron consists of a somatodendritic domain, the axon initial segment (AIS), nodes of Ranvier and presynaptic terminals. The action potential is generated at the AIS, located in the proximal region of the axon and propagates via saltatory conduction down the nodes of Ranvier of a myelinated axon to the presynaptic terminals. Ankyrin-G (red) is considered the master organizer of the AIS and controls localization of membrane-associated proteins such as Kv and Nav channels as well as the cell adhesion molecules NF186 and NrCAM. Nav channels found at the AIS include Nav 1.1, Nav1.2 and Nav1.6. Ankyrin-G is also linked to the underlying actin cytoskeleton through its interaction with βIV-spectrin and to the microtubule cytoskeleton through interactions with EB1/3 proteins. Other components of the AIS include protein kinases CK2, Cdk5 and CAMKII as well as Cav, Kv1 and PSD-93.

Neurons are polarized cells made up of structurally and functionally distinct processes, dendrites and axons, which direct the flow of information throughout the nervous system. The dendrites are often composed of multiple branches and dendritic spines that receive signals from upstream synaptic inputs and transmit this information to the axon. The axon propagates electrical signals, known as action potentials, to downstream neurons by the opening of voltage gated-sodium channels at specialized excitable microdomains referred to as the axon initial segment (AIS) and nodes of Ranvier (Figure 1). Action potential initiation at the AIS and efficient propagation across the nodes of Ranvier requires the localization of high concentrations of voltage-gated ion channels. In addition, the AIS and nodes also contain high densities of cell adhesion molecules and scaffolding proteins that anchor these critical ion channels to the underlying cytoskeleton networks.

Despite similar structural composition between the AIS and the nodes, the mechanisms by which these microdomains form are quite different. The formation of the nodes of Ranvier is strongly influenced by both intrinsic and extrinsic factors, whereas localization of these same proteins to the AIS relies mainly on factors intrinsic to the neuron. The intricate formation and function of excitable axonal microdomains of the vertebrate nervous system plays a critical role in fast neuronal signaling and higher order cognitive processing. Several excellent detailed reviews about the structural organization and physiology of the AIS and nodes of Ranvier have been published (see: Leterrier and Dargent, 2014; Yoshimura and Rasband, 2014; Rasband and Peles, 2015). This review focuses on the recent advances in our understanding of structural and functional mechanisms underlying the formation and function of AIS and nodes of Ranvier and how disruptions in these mechanisms influence neurological health and disease.

## Overview of The Function and Intrinsic Assembly of The AIS

The AIS is a specialized membrane domain approximately 10–60 μm long and is generally located at the most proximal region of the axon (Palay et al., 1968). This domain is characterized by high-densities of voltage-gated ion channels and functions as the gatekeeper of action potential initiation as well as axonal polarity (Kole et al., 2008; Bender and Trussell, 2012; Kole and Stuart, 2012; Jones and Svitkina, 2016; Figure 1). AIS assembly is an intrinsic process within the neuron, in contrast to the formation of nodes of Ranvier, which also require extracellular glial-derived signals. The scaffolding protein ankyrin-G is regarded as the master organizer of the AIS as it coordinates the localization of all known AIS components (Bennett and Baines, 2001; Jenkins and Bennett, 2001; Leterrier et al., 2017). Other critical proteins involved in AIS assembly and function include Nav channels (Kordeli et al., 1995; Zhou et al., 1998), neuronal KCNQ potassium channels (Pan et al., 2006), the cell adhesion molecule neurofascin-186 (NF186; Davis and Bennett, 1994; Jenkins and Bennett, 2001; Ango et al., 2004; Dzhashiashvili et al., 2007), casein kinase II (CK2; Bréchet et al., 2008) and βIV spectrin-actin cytoskeletal proteins (Komada and Soriano, 2002; Yang et al., 2007; Figure 1).

Work from Gary Banker and others with cultured hippocampal neurons has established the nomenclature for stages of neuronal development (Dotti et al., 1988). At the start of neuron development, referred to as stage 1, multiple lamellipodia protrude around the entire circumference of the cell. In stage 2, the lamellipodia progress into several short and identical neurites within 12–24 h of plating. Neuronal polarity begins to develop at 24–48 h in stage 3 during which one of the immature neurites rapidly elongates and subsequently acquires axonal properties. Stage 4 occurs shortly after the formation of the axon, where the remaining immature neurites slowly transition into the dendrites at 3–4 days (Dotti et al., 1988). The AIS first forms in cultured hippocampal neurons between stages 3 and 4 (approximately 3–4 days *in vitro*) indicated by the clustering of ankyrin-G, the first detectable marker of the AIS (Yoshimura and Rasband, 2014). *In utero* electroporation of GFP to label neurons *in vivo* revealed the first noticeable accumulation of ankyrin-G occurs in the proximal axon at approximately P1 after most neurons have migrated to their final destination in layer II/III of the cortex (Galiano et al., 2012). In contrast, Gutzmann et al. (2014) discovered ankyrin-G appears at the proximal axon at embryonic day 14.5 in the visual cortex. Further, analysis of AIS formation *in vivo* using spinal motor neurons, demonstrated that ankyrin-G is first expressed along the length of the axon before gradually becoming restricted to the proximal axon at embryonic day 13.5 (Le Bras et al., 2014). It is not clear whether these findings represent a difference in assembly of the AIS in a brain region-specific or cell type-specific manner (i.e., primary motor cortex vs. visual cortex vs. spinal motor neurons); however, in all cases ankyrin-G is the first resident protein of the AIS to appear.

## Giant Ankyrins Key to Axonal Structure and Function

The vertebrate genome contains three members of the ankyrin gene family: *ANK1*, *ANK2* and *ANK3* (encoding ankyrin-R, ankyrin-B and ankyrin-G, respectively). Alternative splicing is a key mechanism underlying the functional diversity and cellular distribution of ankyrins. In addition to the canonical 190 kDa ankyrin-G, alternative splicing of the giant 7.8-kb exon produces a 270 kDa isoform, which only utilizes the first ~2700 nucleotides of the giant exon due to in-frame splicing, and a giant 480 kDa isoform which utilizes the entire giant exon. Similar alternative splicing of *ANK2* gives rise to a 220 kDa isoform and a 440 kDA isoform of ankyrin-B. The giant ankyrin-G differs from the large isoform of ankyrin-B due to the presence of a 40 kDa serine and threonine rich domain located on the N-terminal side that is modified by O-linked *N*-acetylglucosamine residues with unknown function (Zhang and Bennett, 1996; Vosseller et al., 2006). Interestingly, giant ankyrins are more prevalent in the genome throughout evolution than originally thought, with many bilaterians expressing giant isoforms with variation between species in the site of insertion (Jegla et al., 2016). Between three species that share homologous insertion sites (*Drosophila*, *Ciona intestinalis* and *Stronglyocentrotus purpuratus*), there is no significant sequence homology outside of a composition bias of increased usage of serine and glutamic acid and there is a huge variation in exon size (7.8 kb in vertebrates vs. 13.3 kb in *C. intestinalis* and 27.8 kb in *Drosophila*). Although the *Drosophila* giant splice variants also show some ability to restrict ion channel mobility within the axon (Jegla et al., 2016), more work is necessary to determine the functions of these different giant ankyrins.

In vertebrates, the 480 kDa isoform of ankyrin-G is localized to the AIS and nodes of Ranvier in myelinated axons. Recent studies demonstrated that, of all the *ANK3* isoforms, the giant 480 kDa ankyrin-G is specifically required for the proper localization of voltage-gated Nav channels, KCNQ2/3 channels, NF186 and βIV-spectrin to the AIS (Jenkins et al., 2015). The authors also identified a critical serine residue located within the giant exon, but outside of the canonical binding site of βIV-spectrin, that regulates spectrin localization via a likely phosphorylation-dependent mechanism. Surprisingly, mice lacking the giant 270 kDa and 480 kDa splice variants of ankyrin-G survive through weaning, whereas mice lacking all three main isoforms of ankyrin-G die immediately after birth (Jenkins et al., 2015). Survival of mice lacking the giant splice forms of ankyrin-G may be because of a compensatory increase in expression of the smaller 190 kDa isoform. Further, mice lacking giant ankyrin-G demonstrate severe movement defects and significant reductions in higher order cognitive processing such as working memory and sensory stimulation as compared to control littermates (Jenkins et al., 2015). These findings highlight the critical importance of the giant exon of ankyrin-G for normal neuronal function at both the cellular level and for synchronization of complex brain circuits.

**Figure 2:**
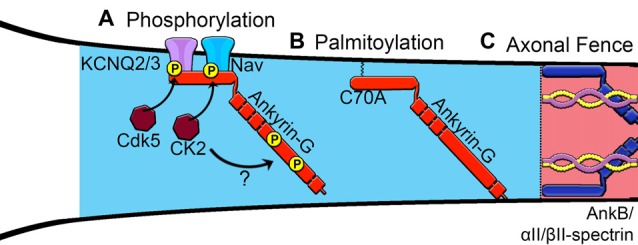
**Proposed mechanisms of assembly of the AIS. (A)** Phosphorylation of Nav channels by protein kinase casein kinase II (CK2) and KCNQ2/3 channels increases affinity for ankyrin-G. Phosphorylation sites within the giant exon of ankyrin-G (red), potentially regulated by CK2, are important for βIV-spectrin binding. **(B)** Palmitoylation of a critical cysteine 70 residue in the membrane-binding domain of ankyrin-G is necessary to target ankyrin-G to the AIS and recruit known binding partners. **(C)** Ankyrin-B interacts with αII-spectrin and βII-spectrin to establish an intracellular barrier or “axonal fence” and maintain ankyrin-G within the proximal axon.

In contrast to the giant splice variant of ankyrin-G, the 440 kDa variant of ankyrin-B is found in unmyelinated axons where it interacts with αII-spectrin and βII-spectrin to establish an intra-axonal barrier that limits ankyrin-G expansion within the axon (Figure 2; Galiano et al., 2012). During postnatal axonal development the majority of the 440 kDa ankyrin-B is replaced by the 220 kDa isoform, lacking the giant insert, which has been shown to be important for the long-range trafficking of cargo down the axon (Lorenzo et al., 2014). Future work will be necessary to fully elucidate the roles of the different splice variants of ankyrin-B and their relationships to ankyrin-G in the proper structure and function of the AIS.

Ankyrins and their spectrin counterparts play a central role in the formation of discrete plasma membrane domains by coordinating the specific subcellular localization of membrane-associated proteins. Ankyrins interact with their membrane-associated proteins through a membrane-binding domain that consists of 24 ANK repeats folded into an extended solenoid structure on the N-terminus (Bennett and Lorenzo, 2013). In canonical ankyrins, the membrane-binding domain is followed by two ZU5 domains, a UPA domain (Wang et al., 2012), a death domain (Wang et al., 2009) and an unstructured C-terminal regulatory domain. The membrane-binding domain of ankyrin-G directly binds to a highly conserved intracellular motif located in the II-III linker domain of Nav channels and to the C-terminus of Kv channels and recruits them to the AIS both *in vivo* and *in vitro* (Pan et al., 2006; Gasser et al., 2012). The cell adhesion molecules NF186 and NrCAM directly interact with the membrane-spanning domain of ankyrin-G through a conserved intracellular five amino acid motif (FIGQY; Zhang et al., 1998; Zhong et al., 2014).

The clustering of ion channels, including Nav and Kv channels, at the AIS is critical for normal neuronal function. The specific subtypes of Nav channels at the AIS include Nav1.1, Nav1.2 and Nav1.6. Early in AIS development, Nav1.2 is the predominant channel found at the AIS; however, as the neuron matures, Nav1.6 becomes the primary channel (Boiko et al., 2003; Osorio et al., 2005). The reason for this shift in Nav channel expression and its physiological impact remains poorly understood. Multiple subtypes of Kv channels have also been identified at the AIS, these include Kv1.1, Kv1.2, Kv1.4, Kv2.1, Kv2.2, Kv7.2 (KCNQ2) and Kv7.3 (KCNQ3), which are important for modulating neuronal excitability (Cooper, 2011).

Deletion of ankyrin-G prevents clustering of other AIS members including KCNQ2/3 channels, NF186, NrCAM, βIV-spectrin and Nav channels in the proximal axon (Zhou et al., 1998; Jenkins and Bennett, 2001; Pan et al., 2006; Jenkins et al., 2015). Genetic deletion of ankyrin-G in mice or silencing ankyrin-G using short hairpin RNA (shRNA) in dissociated neurons results in the loss of Nav channel clustering at the membrane (Zhou et al., 1998; Fache et al., 2004; Hedstrom et al., 2008). The assembly of the AIS through interaction of both the ion channels and cell adhesion molecules to ankyrin-G is regulated by phosphorylation. Interestingly, the phosphorylation of Nav channels is facilitated by protein kinase CK2, which greatly increases Nav affinity for ankyrin-G (Figure 2; Bréchet et al., 2008), whereas phosphorylation of the FIGQY motif on NF186 and NrCAM inhibits interaction with ankyrin-G (Tuvia et al., 1997). The NF186-ankyrin-G-Nav channel protein complex is linked to the underlying actin cytoskeleton through ankyrin-G’s interactions with βIV-spectrin (Berghs et al., 2000).

Spectrins are a group of cytoskeletal proteins that contribute to the mechanical support of axons through direct interaction with ankyrins. βIV-spectrin is a member of the spectrin family, a group of flexible rod-shaped cytoskeletal proteins that exist as tetramers with two α and two β subunits (Ogawa et al., 2006; Uemoto et al., 2007; Galiano et al., 2012). In mammals, although β-spectrins are encoded by five different genes, only βIV-spectrin is found to be enriched at the AIS and nodes of Ranvier, and its recruitment to these sites depends on the direct interaction with ankyrin-G (Yang et al., 2007). Since all β-spectrins contain the canonical ankyrin-spectrin interaction site (Davis et al., 2009), what is unique about βIV-spectrin that allows its clustering at the AIS and nodes of Ranvier? Studies have shown that the first Zu5 domain of ankyrin is the canonical β-spectrin binding site since this is where βII-spectrin binds to ankyrin-B in neonatal cardiomyocytes and where βI-spectrin binds to ankyrin-R in erythrocytes (Mohler et al., 2004; Ipsaro and Mondragon, 2010; Ipsaro et al., 2010). However, the presence of the DAR999AAA mutation in the 480 kDa ankyrin-G, which is known to abolish ankyrin-spectrin binding at Zu5, had no effect on its ability to cluster βIV-spectrin to the proximal axon. In addition, knockout and rescue with the 270 kDa ankyrin-G failed to properly localize βIV-spectrin to the AIS despite the fact that all isoforms of ankyrin-G share the Zu5 domain and that 270 kDa ankyrin-G is capable of interacting with βIV-spectrin in immunoprecipitation experiments (Komada and Soriano, 2002; Hedstrom et al., 2008; Jenkins et al., 2015). Interestingly, mutation of a critical S2417A site found within the giant exon of the 480 kDa ankyrin-G greatly reduces βIV-spectrin localization to the AIS. These findings demonstrate that recruitment of βIV-spectrin to the AIS by ankyrin-G occurs independently of the canonical ankyrin-spectrin binding site in the first Zu5 domain and perhaps offers a novel mechanism as to why βIV-spectrin is localized to the AIS. In addition, the 270 residues between the last spectrin repeat and the PH domain of βIV-spectrin, which are not found in other β-spectrins, may play an important role in the noncanonical recruitment of βIV-spectrin to the AIS. Although this stretch is predominantly unstructured, there are ~70 amino acids that are strongly predicted to form alpha helical coils and this may represent an important interaction surface. More work is necessary to determine the precise mechanisms by which ankyrin-G recruits βIV-spectrin to the AIS.

Despite the fact that βIV-spectrin can interact with αII-spectrin (Uemoto et al., 2007); αII-spectrin has not yet been identified at the AIS. In contrast, βII-spectrin and αII-spectrin assemble with one another in the distal axon (Uemoto et al., 2007). βII-spectrin has been implicated in the initial assembly of the AIS as genetic ablation of βII-spectrin in immature neurons alters βIV-spectrin’s ability to appropriately accumulate at the AIS (Zhong et al., 2014). βII-spectrin becomes enriched in a periodic arrangement in the AIS very early in development, before the other AIS proteins are detectable, and then migrates to more distal regions within the axon. The migration of βII-spectrin down the axon coincides with the appearance of ankyrin-G and βIV-spectrin at the AIS (Zhong et al., 2014). If βII-spectrin is providing initial structural support, what is the function of the clustered βIV-spectrin? One potential role could be to function as the coordinator of a signaling platform for calcium-mediated signaling through calmodulin-dependent kinase II (Hund et al., 2010).

## Potential Mechanisms of Ankyrin-G Recruitment to The AIS

Increasing evidence supports the role of ankyrin-G as the master organizer of the AIS; however, a major unresolved question is how ankyrin-G itself is recruited to the proximal axon to initiate this process (Figure 2). It has been shown that multiple domains of ankyrin-G cooperate with one another to drive its localization to the AIS (Zhang and Bennett, 1998).He et al. (2012) demonstrated that the addition of a fatty acid palmitate, termed S-palmitoylation, to a critical cysteine residue in the membrane-binding domain of the 190 kDa ankyrin-G is necessary for ankyrin-G membrane association and proper polarized localization in epithelial cells. In addition, palmitoylation of the 270 kDa ankyrin-G at the cysteine 70 site is necessary to target ankyrin-G to the AIS and the presence of a C70A mutation in ankyrin-G fails to cluster at the AIS and cannot recruit neurofascin or Nav channels (He et al., 2012). Of the 23 members of the aspartate-histidine-histidine-cysteine (DHHC)-containing protein palmitoylacyltransferases, zDHHC5 and zDHHC8 were identified as the only family members that localize to the lateral membrane and are responsible for the palmitoylation and targeting of ankyrin-G (He et al., 2014). Although the cysteine 70 residue is conserved within all major splice variants of ankyrin-G, it remains unknown which palmitoylacyltransferases localize the giant 480 kDa isoform to the AIS and whether palmitoylation of ankyrin-G occurs specifically at the AIS or within the cell body (Figure 2). Future studies are needed to characterize the palmitoylacyltransferases capable of palmitoylating neuronal ankyrin-G and to evaluate the spatial and temporal regulation of this process during AIS development.

Another important mechanism for the control of AIS formation is phosphorylation of ankyrin-G and its binding partners. Phosphorylation of KCNQ2/3 channels by cyclin-dependent kinase 5 (Cdk5) and phosphorylation of Nav channels by CK2 increase binding affinity to ankyrin-G. Bréchet et al. (2008) showed that CK2 phosphorylation of various serine residues (S1112, S1124 and S1126) and a glutamate residue (E1111) on Nav1.2 regulates Nav channel association with ankyrin-G. These data show that increasing the affinity of ion channels for ankyrin-G is an important regulatory step in the formation of the AIS. Overall, posttranslational modifications are an important step in AIS formation. It will be interesting to see if there are other posttranslational modifications on ankyrin-G and its partners and how these modifications are altered under different signaling conditions.

Lastly, the distal axon cytoskeleton, which is composed of ankyrin-B, αII-spectrin and βII-spectrin complexes, has been proposed to create a boundary that restricts ankyrin-G to the proximal axon (Figure 2). Manipulating the position of this boundary closer to the soma by overexpression of ankyrin or spectrin resulted in a shorter AIS, whereas shifting the boundary away from the soma caused the AIS to become elongated (Galiano et al., 2012). Silencing of ankyrin-B with shRNA inhibits AIS assembly and causes ankyrin-G to distribute throughout the distal axon in cultured neurons (Galiano et al., 2012). In contrast, Lorenzo et al. (2014) observed no gross detectable abnormalities in the AIS in 8 DIV hippocampal neurons derived from ankyrin-B-null mice lacking the 440 kDa and 220 kDa isoforms. Instead, deletion of ankyrin-B results in shortened axonal tracts and impaired axonal transport due to the loss of ankyrin-B association with dynactin and dynein mediated cargo transport (Lorenzo et al., 2014). Elucidating the role of the ankyrin-B/αII-spectrin/βII-spectrin network in the formation of the AIS *in vivo* will be important for the understanding of human diseases involving dysfunction of ankyrins and spectrins. In addition, it remains unclear how 480 kDa ankyrin-G, found both at the AIS and at the distal nodes of Ranvier, is able to avoid the restriction at the proximal axon by the ankyrin-B/spectrin cytoskeletal boundary. It is attractive to speculate that nascent ankyrin-G protein is locally translated at the node of Ranvier or perhaps locally palmitoylated; however, the exact mechanisms controlling AIS ankyrin-G vs. that found at the node of Ranvier remains poorly understood.

## AIS Maintenance and Analysis of Cytoskeletal Composition Through High-Resolution Microscopy

A primary function of the AIS is to maintain polarity of the proximal axon. One potential mechanism is the role of the AIS as a diffusion barrier to inhibit the mobility of membrane-associated proteins from dispersing from one neuronal domain to another. To support this theory, Kobayashi et al. (1992) first suggested the presence of a diffusion barrier at the axonal hillock or AIS after they observed fluorescently labeled phospholipids are static within the axonal membrane in hippocampal cultures, whereas no labeling was observed within the somatodendritic domain. Winckler et al. (1999) then showed that the transmembrane protein L1CAM and the GPI-anchored protein Thy-1 display markedly reduced mobility at the AIS and they may be constrained by a cytoplasmic tether to actin filaments since the disruption of actin caused the proteins to freely distribute between the axonal and somatodendritic compartments. Nakada et al. (2003) further supported these findings by showing that ankyrin-G accumulation in the AIS at 7–10 DIV of developing hippocampal neurons correlates with a dramatic decrease in the rate of phospholipid and Nav channel diffusion.

A more recent study by Song et al. (2009) proposed a second role of the AIS in which ankyrin-G and actin filaments create a selective filter or intracellular sieve within the cytoplasm that blocks the passage of somatodendritic proteins and large macromolecule from entering the axon. For example, axonal motor proteins of the kinesin superfamily (KIFs) were allowed entry into the axon, whereas dendritic cargos and the microtubule-associated protein 2 (MAP2) were found exclusively in the somatodendritic domain of the neuron and become excluded from the AIS throughout the course of assembly (Song et al., 2009). Silencing ankyrin-G expression in hippocampal neurons or genetic deletion of ankyrin-G *in vivo* results in disassembly of the AIS and causes the proximal portion of the axon to acquire dendritic characteristics including dendritic spines and the presence of MAP2 (Hedstrom et al., 2008; Sobotzik et al., 2009). In addition, Jenkins et al. (2015) showed invasion of MAP2 into the axonal process in mice lacking only the giant isoforms 270/480 kDa of ankyrin-G, which indicates that the AIS does play a role in determining the site of axonal specification. Interestingly, however, the axonal process eventually excludes MAP2 and acquires the axonal marker, neurofilament, despite a complete lack of the AIS. These data demonstrate that the AIS is critical for maintaining axonal identity in the proximal axon, but also that a transition from dendritic to axonal character can occur in an ankyrin-G and AIS-independent manner. The exact mechanisms underlying this transition are unknown.

An important prediction of both the diffusion barrier and selective filter models is that dendritic and axonal cargos would randomly mix in the absence of the AIS. Studies evaluating the dendritic-specific cargos, transferrin receptor and TGN38, in neurons completely lacking ankyrin-G showed that these dendritic proteins maintain localization within the dendrites, but were excluded from the distal axon despite the absence of all known AIS components. In addition, the complete loss of the AIS in ankyrin-G-null neurons revealed anterograde and retrograde transport rates of LAMP-1, a relatively large (50–500 nm) lysosome, were indistinguishable between the AIS and distal axon in hippocampal neurons (Jenkins et al., 2015). These findings correspond with other work showing there is no difference in the trafficking of the neuronglia cell adhesion molecule (NgCAM) in the AIS as compared to the distal axon (Petersen et al., 2014). Furthermore, despite the loss of the AIS in total ankyrin-G-null or 480 kDa ankyrin-G-null neurons, MAP2 remains excluded from the distal axon (Jenkins et al., 2015), which suggests neurons may contain a secondary intrinsic property necessary to maintain distal axonal identity. The exclusion of dendritic cargo from the distal axon in cells lacking ankyrin-G is reminiscent of the separation of dendritic and axonal compartments seen before the AIS has been established (Silverman et al., 2001; Nakada et al., 2003; Petersen et al., 2014). Future studies need to evaluate additional AIS-independent mechanisms and how they may be critical to establish and maintain distinct axonal and dendritic polarized compartments.

While this work supports the existence of a diffusion barrier or selective filter that restricts phospholipids, membrane and cytoplasmic proteins and transport vesicles, the molecular composition of the cytoskeletal structure involved in the maintenance of neuronal polarity remains poorly understood. Recent work utilizing light and scanning microscopy showed that dense clusters of actin filaments within the AIS prevented the transport of vesicles that contain dendritic cargo from entering into the axon (Watanabe et al., 2012). Further, live-imaging experiments demonstrated that vesicles containing dendritic cargo enter the axon and dendrites with equal frequency; however, once inside the AIS the vesicles with dendritic proteins reverse directions and proceed toward the somatodendritic domain via an actin and myosin Va-dependent mechanism, whereas vesicles with axonal proteins proceed efficiently down the axon (Al-Bassam et al., 2012). These findings indicate actin filaments may be a key component for the transport of selective axonal cargo; however, there was still a lack in our understanding regarding actin organization within the AIS. Jones et al. (2014) recently sought to evaluate the sophisticated architecture of the AIS cytoskeleton in mature hippocampal neurons using platinum replica electron microscopy (PREM). The results showed an array of microtubule bundles covered in a dense submembranous coat comprised of known AIS proteins including ankyrin-G, βIV-spectrin, neurofascin, Nav channels and actin filaments (Jones et al., 2014). Interestingly, although they failed to identify a dense actin network within the AIS, they discovered subpopulations of actin that alternate between short, stable and longer, flexible filaments.

Recent advances in super resolution microscopy have revealed further insights to the arrangement of the submembranous axonal cytoskeleton and the mechanisms by which the AIS may maintain axonal polarity. A recent study quantitatively sought to determine the nanoscale organization of the AIS using Stochastic Optical Reconstruction Microscopy (STORM). They revealed the actin filaments form “actin rings” that distribute consecutively throughout the entire length of the AIS and are spaced roughly 190 nm apart. In addition, they also determined that ankyrin-B was also found to be periodically localized between the adjacent actin rings in the distal axon (Xu et al., 2013). This unique orientation occurs because βIV-spectrin connects between the adjacent actin rings in a lateral, head-to-head orientation. Further, implementation of 3D-STORM, utilizing antibodies directed against epitopes to either end of ankyrin-G, revealed βIV-spectrin binds periodically on the N-terminus of ankyrin-G, whereas the unstructured C-terminal tail extends ~30 nm internally into the AIS cytoplasm where it may interact with additional cytoplasmic binding partners (Leterrier et al., 2015).

## Axonal Polarization and Vesicle Trafficking

Besides ankyrin-G, βIV-spectrin and actin, what additional cytoskeletal proteins are important to maintain neuronal polarity? There is strong evidence that microtubule-based motor proteins influence the selective filtering of cargo transport into axons and dendrites, but the mechanisms underlying this process is not well understood (Witte et al., 2008; Hoogenraad and Bradke, 2009). Interestingly, Jacobson et al. (2006) showed that the axonal transport protein, kinesin-1, accumulates at a single immature neurite before polarization, which suggests that molecular differences between neurites exist long before the emergence of the axon. The selective transport of signaling proteins to the axon would allow for axon-specific growth from an individual neurite and the timing of this process throughout neuron development would be critical to initiate and maintain axonal polarity. One model suggests the pre-axonal exclusion zone (PAEZ), which is located within the axon hillock, distinguishes the AIS from the soma and may be important for the sorting of somatodendritic and axonal cargo (Farias et al., 2015). Here, the carrier vesicles bind to different microtubule motors that mediate transport either towards the dendrites or down the axon. Organelles that normally bind kinesin-1 or other axonal kinesins can migrate across the PAEZ and down the axon; however, vesicles that bind to dynein or other kinesins are directed to the dendrites.

Another model suggests that, since the microtubules within the AIS are primarily oriented with the plus-ends facing away from the cell body and in the dendrites the microtubules are of mixed orientation, the designated kinesins prefer one orientation over the other driving selective axonal or dendritic trafficking (Hirokawa and Takemura, 2005; Jacobson et al., 2006; Kapitein and Hoogenraad, 2011). In further support of this model, recent work showed that microtubule plus-end binding proteins EB1 and EB3 accumulate in the AIS through direct association with ankyrin-G. Following shRNA-mediated knockdown of ankyrin-G the AIS disassembles and results in a dramatic upregulation in the expression of EB1 and EB2. Thus, it is possible that the C-terminal tail of ankyrin-G extends within the axoplasm to control the proper formation of microtubule bundles and regulate AIS stability. This prediction is consistent with the loss of bundled microtubules seen in the proximal axon of mice lacking ankyrin-G (Sobotzik et al., 2009). Fréal et al. (2016) showed the cooperative interaction between 480 kDa isoform of ankyrin-G and end-binding proteins of the microtubule cytoskeleton drives AIS assembly and axon polarity and suggests another potential mechanism that drives ankyrin-G localization to the AIS.

Kinesins motors are mainly involved in the anterograde transport of dendritic cargo by migrating towards microtubule plus-ends, whereas dynein motors move in retrograde fashion towards microtubule minus-ends within axonal tracts. In support of this view, experiments using fluorescently-labeled mutant kinesin motors were conducted to monitor which subtypes of kinesin motors translocate to either dendritic or axonal domains (Nakata et al., 2011). Kuijpers et al. (2016) discovered that the Nuclear distribution element-like 1 (NDEL1) facilitates dynein activation on somatodendritic cargos that enter the proximal axon and reverses their movement to a retrograde manner. NDEL1 is highly concentrated in the AIS via a direct link with ankyrin-G through its C-terminus tail and with LIS1 through its N-terminus tail. LIS1 has been shown to be an important regulator of NDEL1-based dynein activity at the AIS (Vallee and Tsai, 2006). The knockdown of ankyrin-G, NDEL1, or LIS1 results in the entry of dendritic cargo into the proximal axon, thus these findings suggest a “quick-switch” mechanism for selective vesicle filtering at the AIS. In addition to kinesin and dynein transport, myosin motors have also been implicated to be important for the sorting of cargo between axonal or dendritic polarized domains by interacting directly with different microtubule components (Lewis et al., 2009).

## Electrical Activity and Plasticity at The AIS

**Figure 3:**
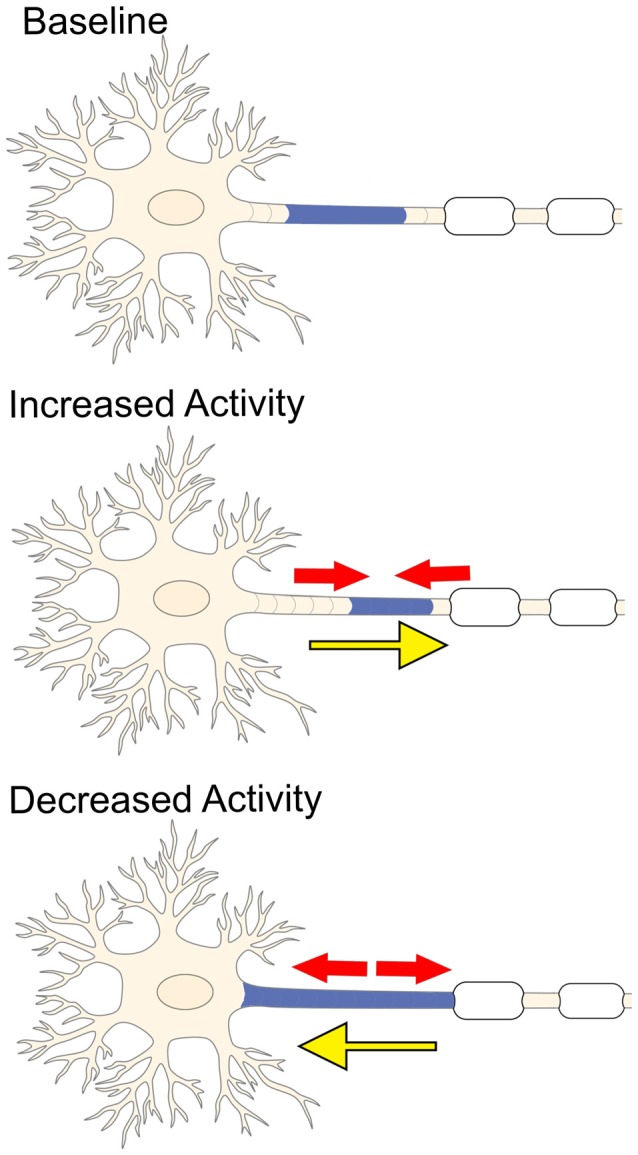
**Electrical activity and plasticity at the AIS.** Increased neuronal activity causes the AIS to shrink (red arrows) and shift more distally away from the soma (yellow arrow). In contrast, inhibiting stimulation of the neuron promotes the AIS to elongate (red arrows) and causes the AIS to shift closer to the soma (yellow arrow).

In vertebrate neurons, the AIS is the site of action potential initiation (Palay et al., 1968; Kole et al., 2008). It has recently been proposed that changes in AIS length, location and/or ion channel expression may occur following varying degrees of neuronal activity (Figure 3; Yamada and Kuba, 2016). This striking plasticity of the AIS arises in an attempt to maintain homeostasis within individual neurons and balance synchronization between complex neuronal circuits. Changes in AIS length were first observed in neurons removed from the avian cochlear nucleus (Kuba et al., 2010). Decreased stimulation of these neurons resulted in elongation of the AIS and an increase in the number of surface Nav channels, which, in turn, enhanced membrane excitability and promoted action potential firing (Kuba et al., 2010). In addition, low-frequency stimulation caused the AIS to shift within about 10 μm of the soma, whereas high-frequency stimulation shifted the AIS about 45 μm away from the soma (Kuba et al., 2006). A similar observation was seen after chronic stimulation of dissociated hippocampal neurons, which also caused a distal shift in the AIS and a corresponding decrease in membrane excitability (Grubb and Burrone, 2010; Evans et al., 2013). These findings suggest changes in electrical activity affect both the dynamics and location of the AIS, but what are the molecular mechanisms that contribute to this plasticity?

Recent studies demonstrated that prolonged depolarization activates L-type and T-type Ca^2+^ channels in pyramidal hippocampal neurons and that the subsequent increase in intracellular Ca^2+^ levels activate Ca^2+^- and calmodulin-dependent protein phosphatases, which may ultimately be responsible for the distal migration and contraction of the AIS (Grubb and Burrone, 2010; Evans et al., 2013, 2015; Muir and Kittler, 2014). Increased intracellular Ca^2+^ has also been shown to activate cyclin-dependent kinase 5 (cdk5) in olfactory bulb dopaminergic interneurons. In addition, heightened cdk5 activity extended AIS length by as much as 100% in mushroom body neurons of *Drosophila*, while depleting cdk5 causes the AIS to significantly shrink or disappear altogether (Trunova et al., 2011). To date, the majority of studies that evaluated activity-dependent AIS plasticity and the underlying cellular mechanisms were conducted in fixed cells and these changes were analyzed amongst a population of neurons. The development of innovative tools capable of labeling the AIS for live-imaging experiments will be beneficial to examine AIS plasticity in individual neurons both *in vitro* and *in vivo*. Recently, Dumitrescu et al. (2016) used a construct consisting of the intracellular domain of a voltage-gated sodium channel Nav1.2 fused to a yellow-fluorescent protein (YFP-NavII-III) to examine the AIS in live neurons. This construct localized specifically to the AIS of dentate granule cells (DGCs) in dissociated hippocampal cultures as well as accurately demonstrated both baseline and activity-induced plasticity changes of the AIS without altering intrinsic neuronal excitability (Dumitrescu et al., 2016). This construct may be useful in future studies to investigate AIS plasticity in individual neurons. Live-imaging the AIS in individual neurons will reduce cell-to-cell and experimental heterogeneity and provide greater insight to the physiological impact underlying changes in AIS size and position.

Relatively little is known about what happens to the ankyrin-spectrin protein networks during periods of AIS plasticity and the physiological impact these changes have on action potential initiation. Post-translational modifications, such as phosphorylation of Nav channels by CK2 or palmitoylation of ankyrin-G and NF186, may be involved in this process (Figure 2; Ren and Bennett, 1998; Bréchet et al., 2008; He et al., 2012). In addition, the changes in the expression levels of ankyrin could underlie AIS plasticity. In dissociated hippocampal cultures, the overexpression of ankyrin-G was shown to elongate the length of the AIS, whereas overexpression of the distal axon cytoskeletal protein ankyrin-B shortened the AIS (Galiano et al., 2012). While phosphorylation is necessary to assemble and maintain proper AIS structure and function, the Ca^2+^-dependent cysteine protease calpain works in opposition to promote the degradation and disassembly of the AIS. Calpain activates the proteolysis of the axonal cytoskeletal proteins including ankyrin-G and βIV-spectrin in the proximal axon and ankyrin-B, αII-spectrin and βII-spectrin in the distal axon (Harada et al., 1997; Czogalla and Sikorski, 2005; Bevers and Neumar, 2008). In addition, Schafer et al. (2009) showed that pharmacological inhibition of calpain was sufficient to attenuate degradation and maintain the molecular organization of the AIS both *in vitro* and *in vivo*. It is also possible that the shortened and distal translocation of the AIS is caused by an overload of Ca^2+^ levels and the subsequent potentiation of calpain-mediated proteolysis.

What are the physiological impacts these changes in AIS structure and position may have on action potential generation? Jenkins et al. (2015) recently showed that mice lacking the giant isoforms (270/480 kDa) of ankyrin-G, and thus all other known components of the AIS, were still able to fire current-induced action potentials with modest effects on action potential frequency and dynamics. One possibility for this phenomenon is that the small 190 kDa ankyrin-G, which displayed a four- to five-fold increase in expression in the giant-exon null mice, is capable of compensating for the 480 kDa ankyrin-G and rescuing Nav channel localization to the plasma membrane. Another possibility is that the action potentials might be generated by Nav channels clustered locally in the somatodendritic domain (Lai and Jan, 2006). If the AIS is dispensable for action potential generation with only minor deficits, what is the evolutionary advantage of having an AIS? One potential answer to this question comes from the abnormalities seen in the gamma oscillations from the EEG recordings of the giant ankyrin-G knockout mice. Gamma oscillations arise through the activity of cortical GABAergic interneurons, which synapse directly on to the AIS and soma of pyramidal neurons and are essential for the proper synchronization of the cortical network (Somogyi, 1977; Markram et al., 2004; Bartos et al., 2007). The AIS thus provides a defined physical location to allow interneurons precise temporal and spatial modulation of action potentials.

## Nodes of Ranvier

**Figure 4:**
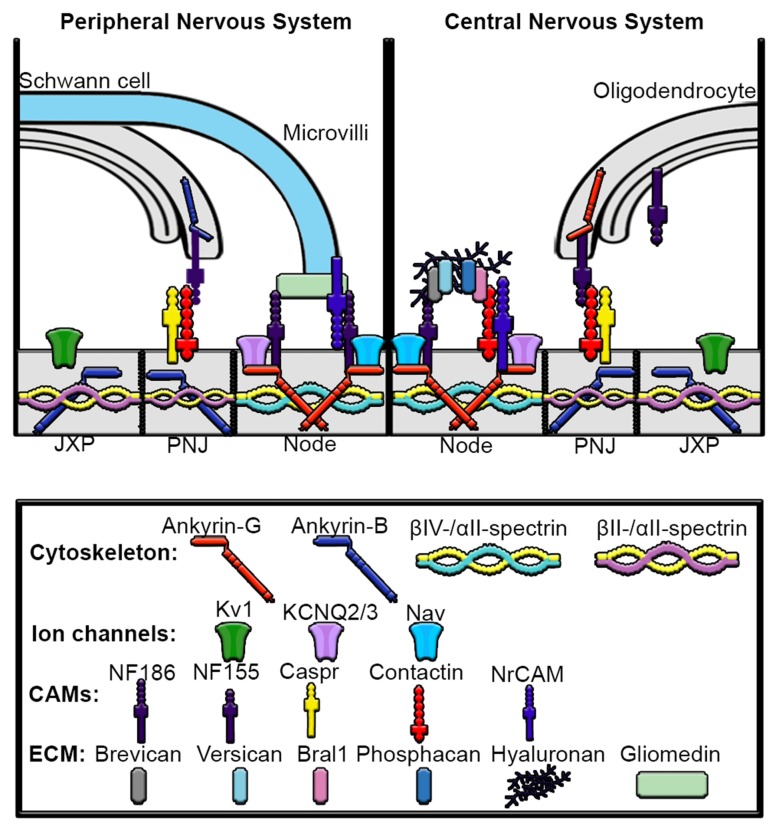
**Mechanisms underlying assembly of peripheral nervous system (PNS) and central nervous system (CNS) nodes of Ranvier.** Axons are myelinated by Schwann cells in the PNS and oligodendrocytes in the CNS. The nodes are gaps in myelinated sheaths and are sites of action potential regeneration. In both the PNS and CNS the node contains high densities of Nav and KCNQ2/3 channels, ankyrin-G, βIV-spectrin, αII-spectrin, NF186 and NrCAM. In the PNS, gliomedin from the microvilli of myelinating Schwann cells directly interacts with NF186 and NrCAM at the node. In contrast, the node of the CNS contains an extracellular matrix (ECM) complex made up of chondroitin sulfate proteoglycans brevican, versican and phosphacan, which interact with contactin, Bral1, hyaluronan and NF186. The paranodal junction (PNJ) flanks the nodes of Ranvier and is the site of Schwann cell contact in the PNS and oligodendrocyte contact in the CNS. In the PNS, Ankyrin-B interacts with NF155 in Schwann cells, which then binds to contactin to connect the myelinating Schwann cell with the axon, whereas in the oligodendrocytes of the CNS, ankyrin-G associates with NF155. Within the paranodal axolemma, ankyrin-B binds βII and αII-spectrin cytoskeleton complexes that play important roles in maintaining paranode barriers. The juxtaparanodes (JXP) are characterized by high-density clustering of Kv1 channels as well as ankyrin-B and βII-spectrin and αII-spectrin tetramers.

Following initiation at the AIS, action potentials must travel rapidly across long distances down the axon in order to reach the synapse. In myelinated axons, action potential regeneration occurs at the nodes of Ranvier, which are gaps between myelin sheaths characterized by very high densities of Nav channels (Figure 1). The molecular composition of the nodes of Ranvier and the AIS are very similar in that they both consist of similar ion channels, cell adhesion molecules and scaffolding proteins (Figure 4; Rasband, 2010). In contrast to the AIS, which is regulated solely by axonal intrinsic signaling, the proper assembly and function of nodes depend on both intrinsic and glial-derived extrinsic mechanisms.

## Molecular Organization of The Nodes of Ranvier

The complex organization of the Nodes of Ranvier is accomplished in part by myelinating Schwann cells in the peripheral nervous system (PNS) and oligodendrocytes in the central nervous system (CNS). The clustering of Nav channels to the node is critically important for the rapid, saltatory propagation of action potentials. Myelination divides the axonal membrane into distinct domains including nodes of Ranvier, paranodes, juxtaparanodes and internodes. The nodes of Ranvier are the sites of action potential repolarization and depolarization due to the clustering of high concentrations of ion channels, including Nav and Kv channels. The complement of Nav and Kv channels at the node are diverse and can include Nav1.1, Nav1.2, Nav1.6, Nav1.7, Nav 1.8 and Nav1.9 which interact with β-subunits Navβ1, Navβ2 and Navβ4 (Fjell et al., 2000; Chen et al., 2002; Boiko et al., 2003; Henry et al., 2005; Duflocq et al., 2008; Black and Waxman, 2012). β2-subunits covalently bond with Nav channels via an extracellular disulfide bond and regulate their surface expression (Chen et al., 2012). Kv channels at the node include Kv3.1b, KCNQ2 and KCNQ3 (Cooper, 2011). In addition to ion channels, ankyrin-G and βIV-spectrin scaffolding proteins are also highly concentrated at the nodes and, similar to the AIS, anchor the ion channels and cell adhesion molecules NF186 and NrCAM to the underlying cytoskeleton network. Recent studies using Stimulated Emission Depleted (STED) microscopy demonstrated a periodic organization of ankyrin-G and βIV-spectrin with the underlying microtubule cytoskeleton at the nodes of Ranvier, similar to that seen in the AIS (D’Este et al., 2015). The paranode flanks the node of Ranvier and is the site where myelinating glial cells form septate-like junctions with the axonal membrane. Ankyrin-G has been shown to be highly enriched within oligodendrocytes on the glial side of the paranodal junction, whereas ankyrin-B is highly expressed at the Schwann cell paranodal membrane (Chang et al., 2014). Glial ankyrins bind to the cell adhesion molecule NF155 at the paranodal junction and contribute to the assembly and maintenance of nodes of Ranvier in both the CNS and PNS. Thus, mutations within *ANK2* or* ANK3* may lead to abnormalities in the AIS and axonal nodes of Ranvier as well as the paranodes within glia. The juxtaparanodes flank the paranodes and are enriched with dense populations of Kv channels known to module action potential conduction and help maintain internodal resting potential. Finally, the internodes make up the majority of the axon and are found underneath the myelin sheaths.

## Assembly of The PNS Nodes of Ranvier

Although the molecular composition between PNS and CNS nodes of Ranvier are similar, the mechanisms involved in their assembly are different mainly due to the glial cells types involved in myelination (Figure 4). In PNS node assembly, Nav channels are initially clustered at the edges of developing myelin sheaths, referred to as the heminodes, by the extracellular matrix (ECM) molecules gliomedin and neuronal cell adhesion molecule (NrCAM) from Schwann cell microvilli interacting with axonal NF186 (Lambert et al., 1997; Eshed et al., 2005; Schafer et al., 2006; Feinberg et al., 2010). Secondly, Nav channels are restricted to the nodal gap by the paranodal junction, which consists of glial-derived NF155, found at paranodal region, in conjunction with Caspr and contactin within the axonal membrane. The interaction between NF155 and the Caspr-contactin complex mediates Schwann cell interaction with the axon and formation of the paranodal junction. These paranodal junctions are thought to act as a restriction barrier during node of Ranvier assembly as the nodes are fully capable of forming in NrCAM and gliomedin knockout-mice, despite the fact that NF186 fails to localize to the heminodes of these mice (Feinberg et al., 2010). In addition, the paranodal junction between myelinating Schwann cells of the PNS (and oligodendrocytes in the CNS) may function as a diffusion barrier to prevent the lateral movement of ion channels along the axonal plasma membrane (Rasband et al., 1999; Pedraza et al., 2001). In contrast, the significance of a diffusion barrier remains controversial since disturbing the paranodal junction only slightly perturbed Nav clustering (Bhat et al., 2001; Thaxton et al., 2011). Interestingly, Amor et al. (2017) recently showed that the paranodal junctions are sufficient to cluster Nav channels to the node of Ranvier in peripheral sensory neurons and retinal ganglion cells of knockout mice deficient of nodal NF186. Further, the authors demonstrate that βII-spectrin plays a role as a diffusion barrier within the paranodal junction to mediate Nav clustering at the node. These findings suggest that the intact paranode can function as a secondary mechanism for Nav nodal clustering independent of axonal NF186 localization by glial-derived proteins.

In addition to gliomedin, other ECM proteins involved in heminode formation include syndecans, laminins, NG2 and versican, all of which also directly interact with NF186 (Occhi et al., 2005). Additional proteins unique to the PNS nodal microvilli are exrin, radixin, moesin, EBP50, dystphin and utophin (Occhi et al., 2005). The paranodal junction then constrict leading to stabilization of the node by NF186 association with ankyrin-G, which subsequently interacts with and recruits Nav channels, Kv channels and βIV-spectrin. Nav and Kv channels bind with high affinity to the membrane-binding domain of ankyrin-G at the node via a CK2 phosphorylation-dependent mechanism as seen in the AIS (Wang et al., 2014; Xu and Cooper, 2015). Recent studies by Ho et al. (2014) discovered that in the absence of ankyrin-G, Nav channels are still clustered to the node of Ranvier by compensation of ankyrin-R and its binding partner βI-spectrin in peripheral sensory neurons and retinal ganglion cells. However, the ability of ankyrin-R to compensate for ankyrin-G at the node of Ranvier remains controversial (Saifetiarova et al., 2017).

## Assembly of CNS Nodes of Ranvier

Similar to the PNS, glial-derived extrinsic mechanisms contribute to CNS formation; however, in contrast to the microvilli of Schwann cells that make contact to the node in the PNS, the oligodendrocytes do not directly interact with the nodes in the CNS. Three important components have been proposed to be important for node of Ranvier assembly in the CNS (Figure 4). First, an ECM complex produced by glial cells promotes NF186 to cluster at the node. The ECM in the CNS contains the chondroitin sulfate proteoglycans brevican, versican, neurocan and phosphacan in addition to tenascin-R, BRal1 and NrCAM. The glial-derived ECM directly interacts with the axonal cell adhesion molecules NF186, NrCAM, contactin-1 and the β-subunit of sodium channel and are likely involved in the long-term maintenance of CNS nodes (Weber et al., 1999; Xiao et al., 1999; Oohashi et al., 2002; Bekku et al., 2009; Dours-Zimmermann et al., 2009; Susuki et al., 2013). Secondly, the paranodal axo-glial complex forms, which consists of three main cell adhesion molecules: neurofascin 155 kDa isoform (NF155) derived from glial cells, and Caspr (contactin-associated protein) and contactin which are generated in the neuron. Lastly, the axonal scaffolding protein ankyrin-G is necessary to cluster and stabilize Nav channels to the node (Gasser et al., 2012). Deletion of the giant splice variants of ankyrin-G resulted in an 80% loss in the number of nodes of Ranvier *in vivo* and the remaining nodes of the corpus callosum were malformed and elongated (Jenkins et al., 2015). Interestingly, while the remaining nodes lacked 480 kDa ankyrin-G and NF186, βIV-spectrin and Nav channels were still present and NF155 persisted at the paranode. Nav channels were clustered at the node, likely due to the dramatic upregulation seen in the 190 kDa isoform of ankyrin-G (Jenkins et al., 2015).

Ankyrin-G is referred to as the master organizer of the AIS; however, because the nodes require extrinsic regulation for their proper formation and function, the role of ankyrin-G as the master organizer of the node of Ranvier is less clear. The fact that ankyrin-G contains binding sites for all known nodal components supports the theory that ankyrin-G is necessary and sufficient for node formation (Hill et al., 2008; Gasser et al., 2012). In addition, mutation of the ankyrin-G-binding domain in NF186 inhibits its ability to cluster at the node (Susuki et al., 2013). Zonta et al. (2008) demonstrated that genetic deletion of both isoforms of NF186 and NF155 completely disrupted nodal and paranodal complexes; however, the authors show that rescue with either NF186 or NF155 independently can promote the assembly of the nodal complex and recruit Nav channels. Since Nav channels, their β subunits and ankyrin-G can interact with NF186 directly, it may not be a surprise that the addition of NF186 is capable of rescuing the node and may even subsequently promote more delivery of NF186. Rescuing with NF155 is more intriguing as NF155 is not found at the node with ankyrin-G or Nav channels, but is still sufficient to rescue assembly of the node (Zonta et al., 2008). Zhang et al. (2015) recently discovered a third isoform of neurofascin, NF140, which is highly expressed early in embryonic development and is capable of clustering Nav channels to the developing node of Ranvier independently of NF186 and NF155. Future research should expand on these findings to better understand how deletion of ankyrin-G or neurofascin disrupts Nav clustering throughout CNS, and how this loss of Nav channels at the node impacts brain function.

While the pioneering work on the AIS and nodes of Ranvier done in cultured cells *in vitro* has given us great insights into the formation and function of these critical subcellular domains, recent work has highlighted the need to examine these mechanisms *in vivo* (Komada and Soriano, 2002; Sherman et al., 2005; Zonta et al., 2008; Susuki et al., 2013; Chang et al., 2014; Jenkins et al., 2015; Amor et al., 2017; Saifetiarova et al., 2017). Specific knockout animal models have elucidated how the AIS and nodes of Ranvier are formed in the intact organisms and have supported many of the findings from *in vitro* studies. Importantly, animal models also give us the ability to examine whether the mechanisms are conserved between cell types. For example, much of the work on the mechanisms of CNS node of Ranvier formation has been done in spinal cord or optic nerve. Are these mechanisms conserved in myelinated axons in the brain?

## Axonal Domain Proteins in Disease and Injury

An increasing number of studies have shown that genetic mutations in components of both the AIS and nodes of Ranvier are involved in the pathophysiology of multiple diseases and injuries. As previously mentioned, ankyrin-G is absolutely essential to maintain the structural composition of the AIS and nodes of Ranvier and for normal axonal polarity. Thus, mutations or loss-of-function of *ANK3* might be expected to have a profound effect on neurological function. Consistent with this idea, genome-wide association studies have identified *ANK3* as one of the most significant risk loci for bipolar disorder, and to a lesser degree schizophrenia (Ferreira et al., 2008; Muhleisen et al., 2014; Roussos and Haroutunian, 2014). Post-mortem brains of schizophrenic patients revealed a 15%–20% decrease in ankyrin-G expression at the AIS of pyramidal neurons in the superficial cortical layer as compared to neurotypical controls, while no significant changes in AIS length were observed (Cruz et al., 2009). A recent study by Lopez et al. (2016) showed that the presence of a bipolar disorder-associated variant in *ANK3* results in reduced expression of the *ANK3* exon 1b isoform in the AIS of parvalbumin-positive (PV) GABAergic interneurons. Interestingly, mice lacking the exon 1b isoform loose Nav channel clustering at the AIS of PV interneurons and demonstrate behavioral characteristics of bipolar disorder, epilepsy and sudden death (Lopez et al., 2016). In addition, *de novo* missense mutations in *ANK3* have been identified in autistic patients as well as severe cognitive deficits, borderline intelligence, severe attention deficit hyperactivity disorder (ADHD) and sleeping problems (Awadalla et al., 2010; Hamdan et al., 2011; Talkowski et al., 2012). The presence of a homozygous premature stop codon predicted to abolish the 480 kDa isoform of ankyrin-G resulted in dramatic cognitive dysfunction and intellectual disability with IQ values below 50 (Iqbal et al., 2015). It will be important to elucidate the precise effects of *ANK3* mutations on neuronal function.

Mutations in voltage-gated sodium channel α subunits and their associated β subunits found at the AIS and nodes of Ranvier have a wide range of profound neurological effects, including epilepsy, neurodegeneration and sudden death. For example, mutations in *SCN1A* (Nav1.1) are associated with Dravet syndrome, a severe myoclonic epilepsy of infancy, as well as West syndrome, genetic epilepsy with febrile seizures plus (GEFS+) and others (Steinlein, 2014). *SCN2A* (Nav1.2) and *SCN8A* (Nav1.6) mutations are found in patients with early infantile epileptic encephalopathy (Steinlein, 2014; Wagnon and Meisler, 2015). Mutations in the sodium channel β subunits are associated with multiple neurological disorders, including GEFS+, Dravet syndrome and neurodegenerative disease (O’Malley and Isom, 2015). In addition to mutations in sodium channel genes, loss-of-function mutations in both KCNQ2 and KCNQ3 potassium channel genes are linked to benign familial neonatal convulsions (Singh et al., 2003).

Disruptions in spectrin cytoskeletal function and assembly have also been associated with neurological disease. The human spectrin family consists of two alpha- and five beta-spectrin subunits, which form heterodimers that assemble into tetramers through head-to-head and lateral associations (Bennett and Lorenzo, 2013). Human dominant in-frame duplications and deletion mutations in *SPTAN1* have been found in patients with early-onset epileptic encephalopathies, hypomyelination, intellectual disability and blindness starting in children under age 3 (Saitsu et al., 2010; Nicita et al., 2015). Mutations in βIII-spectrin, which is highly expressed in cerebellar Purkinje neurons, have been associated with spinocerebellar ataxia type 5 (Ikeda et al., 2006).

Increasing evidence also suggests degeneration of the axon is an important component underlying multiple sclerosis (MS) pathology; however, the mechanisms that contribute to axonal loss remain elusive (Dutta and Trapp, 2007). Patients suffering from MS demonstrated changes in expression and localization of Nav channels and neurofascin, as well as the paranodal protein Caspr (Wolswijk and Balesar, 2003; Craner et al., 2004; Coman et al., 2006; Howell et al., 2006). One potential mechanism that contributes to MS may be abnormal axo-glial interaction at the paranode, which would be expected to disrupt axonal transport and alter normal organization of myelinated axons (Sousa and Bhat, 2007). Mathey et al. (2007) identified autoantibodies from MS patients that specifically target the extracellular domains both axonal NF186 and glial NF155, disrupt conduction and ultimately lead to axonal injury that mimics the pathology of MS. In addition to the nodes of Ranvier, the effect of demyelination on the AIS may be another potential mechanism that contributes to MS.Hamada and Kole (2015) showed that demyelinating axons using cuprizone caused the AIS to shift more proximal to the soma and reduced action potential initiation. However, they observed no changes in ankyrin-G, βIV-spectrin and Nav1.6 expression at the AIS following demyelination (Hamada and Kole, 2015). Consistent with these findings, Clark et al. (2016) also found AIS components remained intact following cuprizone-induced demyelination. In contrast, the authors discovered the proper clustering of ankyrin-G, βIV-spectrin and Nav1.6 was lost at the AIS of mice after chronic exposure of experimental autoimmune encephalomyelitis (EAE), an inflammatory model of MS (Clark et al., 2016). Ultimately, the AIS is a primary target during inflammation and, in addition to demyelination of the distal axon, may contribute to inflammatory demyelinating diseases such as MS.

In a rat model of mild traumatic brain injury, Baalman et al. (2013) showed that exposure to a single blast wave results in long-term changes in memory within these rats and, at the cellular level, significant decreases in AIS length. These changes in the AIS perhaps highlight a potential mechanism underlying mild traumatic brain injury and future studies will be important to elucidate the specific molecular components that contribute to the structural and functional changes in the AIS.

Overall, changes in excitable domains of the axon or their constituent proteins have profound impact on neurological function. Although many of the proteins of the AIS and nodes of Ranvier have important functions in other cellular domains, the overlapping phenotypes seen with loss of function of different AIS and nodal components suggest that dysfunction of these axonal membrane domains is a major factor in the development of disease. As we increasingly understand the genetic basis of neurological disorders, we will likely uncover more genes involved in the formation and function of axonal domains that can give us more insight into the etiology of human disease.

## Conclusion

The structural assembly and maintenance of the axon relies on the precise organization between ankyrins, spectrins, membrane-associated proteins and actin and microtubule cytoskeletal proteins. The mechanisms underlying the interaction between these components at the AISs and nodes of Ranvier are now becoming more apparent. A better understanding of the organization and maintenance of axonal excitable domains as well as how abnormalities in their signaling may lead to altered axonal function will provide insight to novel therapeutic targets for the treatment of human diseases of the nervous system.

## Author Contributions

ADN and PMJ wrote the manuscript.

## Conflict of Interest Statement

The authors declare that the research was conducted in the absence of any commercial or financial relationships that could be construed as a potential conflict of interest.
